# Gene dynamics of haplodiploidy favor eusociality in the Hymenoptera

**DOI:** 10.1111/evo.14518

**Published:** 2022-06-06

**Authors:** Jack da Silva

**Affiliations:** ^1^ School of Biological Sciences University of Adelaide Adelaide South Australia Australia

**Keywords:** altruism, eusociality, haplodiploidy, helper, Hymenoptera, worker

## Abstract

The problem of whether haplodiploidy is responsible for the frequent evolution of eusociality in the Hymenoptera remains unresolved. The little‐known “protected invasion hypothesis” posits that because a male will transmit a new allele for alloparental care to all his daughters under haplodiploidy, such an allele has a higher probability of spreading to fixation under haplodiploidy than under diploidy. This mechanism is investigated using the mating system and lifecycles ancestral to eusocial lineages. It is shown that although haplodiploidy increases the probability of fixation of a new allele, the effect is cancelled by a higher probability of the allele arising in a diploid population. However, the same effect of male haploidy results in a 30% lower threshold amount of reproductive help by a worker necessary to favor eusociality if the sex ratio of dispersing first‐brood offspring remains even. This occurs because when first‐brood daughters become workers, the sex ratio of dispersing first‐brood offspring becomes male‐biased, selecting for an overall female‐biased first‐brood sex ratio. Through this mechanism, haplodiploidy may favor eusociality in the absence of a female‐biased sex ratio in dispersing reproductive offspring. The gene‐centric approach used here reveals the critical role of male haploidy in structuring the social group.

The role of haplodiploidy in the frequent evolution of eusociality in the aculeate Hymenoptera (ants, bees, and wasps) remains unresolved. Hamilton (1964a), in developing inclusive fitness theory, argued that the close genetic relatedness of full sisters under haplodiploidy (because they share their father's haploid genome) would favor the evolution of effectively sterile helpers that preferentially raise sisters rather than their own offspring (Hamilton 1964b). However, since brothers are more distantly related to sisters (brothers are haploid, inheriting a single copy of the mother's genome), with an even sex ratio, the inclusive fitness gain from raising full siblings is equal to that of raising offspring (Trivers and Hare 1976). And biasing the sex ratio of reproductive offspring produced with help toward females does not necessarily resolve the issue, since then males have higher reproductive value (mating success), which selects for a more even sex ratio (Craig 1979, 1980). Several hypotheses have been proposed for how haplodiploidy may nevertheless have favored the origin of eusociality, as opposed to acting after eusociality was established such as through worker reproduction or worker manipulation of sex ratios (e.g., Crozier 1977; Alpedrinha et al. 2013; Alpedrinha et al. 2014; Rautiala et al. 2019). These may be divided into two types: mechanisms that produce different sex ratios among broods or nests in noneusocial species (“split sex ratios”) (Seger 1983; Grafen 1986; Gardner et al. 2012) and mechanisms that concentrate closely related females within a nest (Reeve 1993; Fromhage and Kokko 2011; Johnstone et al. 2012). Mechanisms causing split sex ratios do not appear to have been common enough to have influenced the origin of eusociality (Gardner et al. 2012; Alpedrinha et al. 2013), and mechanisms causing the concentration of close relatives do not appear to have been fully developed or tested.

The little‐known “protected invasion hypothesis” posits that haplodiploidy increases the probability of fixation of a new dominant mutation causing alloparental care (Reeve 1993). The effect is due to all daughters of a male carrying the mutation inheriting the mutation if the male is haploid, but only half of the daughters inheriting the allele if he is diploid. Here, this effect is examined using the lifecycles of subsocial Hymenoptera, which are ancestral to eusocial lineages. It is shown that although the threshold amounts of help necessary for a new allele for reproductive altruism to be favored are the same under haplodiploidy and diploidy, as derived from inclusive fitness accounting, the probably of fixation of the allele is higher under haplodiploidy. Surprisingly, however, this effect of haplodiploidy is cancelled by the higher probability of a mutation arising in a diploid population. Rather, when considering the implications of the spread of an altruism allele for the sex ratio of dispersing offspring, and consequently, adjusting the sex ratio of first‐brood offspring to ensure an even sex ratio of dispersing offspring, haplodiploidy requires a 30% lower threshold of help necessary for eusociality to be favored. Thus, haplodiploidy may favor the evolution of eusociality without a female biased sex ratio in reproductive offspring.

## Methods

### SUBSOCIAL LIFECYCLES

**Figure 1:**
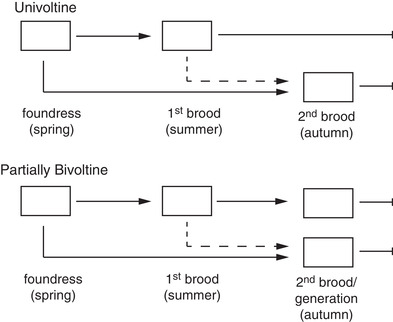
Lifecycles. Boxes represent females; only females are shown for simplicity. Solid arrows represent reproduction and dashed arrows represent helping. Lines terminated with a bar indicate diapause.

The basic approach is to count the number of copies of an allele for altruism in dispersing offspring produced by a mated pair of individuals at the end of an annual breeding season. Results depend on the mating system and lifecycle because these determine how fitness is calculated. Specifying the life histories relevant to the origin of eusociality in the Hymenoptera also permits theory to be more easily tested empirically. Monogamy is assumed, as this mating system is ancestral for eusocial lineages of Hymenoptera (Hughes et al. 2008). The lifecycles of subsocial bees and wasps closely related to eusocial lineages and primitively eusocial species are either univoltine or partially bivoltine (Fig. 1). With a univoltine lifecycle, a nest foundress produces two broods, one in the spring and one in the autumn. Daughters from both broods disperse, mate, and enter diapause before winter. All sons disperse, mate, and die without entering diapause. The foundress dies before winter. This lifecycle has been observed for primitively eusocial paper wasps (*Polistinae*) (Reeve et al. 1998) and sweat bees (*Halictinae*) (Yanega 1988; Schwarz et al. 2007). The only difference with a partially bivoltine lifecycle is that first‐brood offspring disperse, mate, and produce their own broods in autumn. This second generation of individuals, emerging in the autumn, disperse and mate, with females then entering diapause and males dying. Their first‐brood parents all die before winter. This lifecycle has been observed for subsocial and primitively eusocial sweat bees (Seger 1983; Schwarz et al. 2007; Danforth et al. 2019). Lifecycles in which both sons and daughters enter diapause as unmated adults give the same results. Some subsocial and primitively eusocial carpenter bees (*Allodapinae*) exhibit these lifecycles (Schwarz et al. 2007; Danforth et al. 2019).

### POPULATION GENETIC MODEL

The simplest possible model is used. It is assumed that helping behavior is determined by a single locus with two alleles, a wild‐type allele, *A*_0_, and an allele for reproductive altruism, *A*_1_, conditionally expressed in first‐brood daughters, the only individuals with an opportunity to help the foundress raise the second brood. The foundress produces two broods of *n* offspring each. With a univoltine lifecycle, a first‐brood daughter may either enter diapause or help her mother produce an additional *b* offspring in the second brood if the daughter carries the altruism allele. With a partially bivoltine lifecycle, a first‐brood daughter may either disperse to produce a brood of *n*_2_ offspring or help her mother produce an additional *b* offspring in the second brood.

### INVASION ANALYSES

Two approaches are taken in tracking allele copies. First, because its simplicity helps elucidate the mechanism, a single copy of the altruism allele is introduced in a mated pair and the number of copies of the allele in dispersing offspring at the end of a single breeding season is counted. To determine the change in the number of altruism allele copies relative to the change in the number of wild‐type allele copies, this count is compared to the number of altruism alleles produced if they were neutral, that is, had no effect on helping behavior and, thus, are indistinguishable from a wild‐type allele. This is referred to as an invasion analysis.

### FULL TIME COURSE ANALYSES

The second approach involves using deterministic recursion equations to track allele frequencies in infinite populations over multiple generations. This is referred to as a full time course analysis. Recursion equations give genotype frequencies in the next generation as a function of the genotype frequencies in the current generation and the fitnesses of mated pairs with different genotypes. The fitness of a mated pair is used because although mating is assumed to be random, gamete fusions producing dispersing individuals are not random because of biases in the genotypes carried by dispersing individuals. The fitness of a mated pair is the number of dispersing offspring at the end of the breeding season. A further issue is that because some first‐brood females act as helpers and do not disperse, the sex ratio of dispersing individuals will become progressively male biased as the altruism allele spreads in the population. This is assumed to be countered by selection for an optimal Fisherian even sex ratio of dispersing individuals at the population level (assuming equal investment in the sexes) (West 2009). This selection is implemented by adjusting the sex ratio of the first brood to become female biased to the extent that the sex ratio of *dispersing* offspring is even. The adjusted sex ratio, in terms of the proportion of offspring that are female, is $f\;=\frac{1}{2-a}\;$, where *a* is the proportion of first‐brood females that act as helpers. For example, if $a\;=\frac{1}{2}\;$, then $f\;=\frac{2}{3}\;$ and, thus, $af\;=\frac{1}{3}\;$ of offspring are female helpers, $f-af\;=\frac{1}{3}\;$ are dispersing females and $1-f\;=\frac{1}{3}\;$ are dispersing males. Details of full time course analyses are provided in Supporting Information, and computer code is archived in the Zenodo digital repository (da Silva 2022).

## Results

### INVASION ANALYSES

#### Univoltine lifecycle

I start with the simpler approach of introducing a single copy of the altruism allele and the simplest conditions of a univoltine lifecycle and a dominant allele. These results are described in detail to demonstrate the approach; results for other conditions are summarized only.

##### Haplodiploidy

With haplodiploidy, there are two possible crosses between a mated pair in which one of the pair carries the allele: either the foundress carries the allele, *A*_0_ *A*_1_ × *A*_0_, or the male carries the allele, *A*_0_ *A*_0_ × *A*_1_. These crosses each produce two broods, each with an even sex ratio. If the foundress carries the allele, in the first brood, because the allele is dominant, half of the daughters (*n*/4) have an *A*_0_ *A*_1_ genotype and these act as helpers (Table 1). The remaining daughters, with genotype *A*_0_ *A*_0_, disperse to mate and enter diapause. Therefore, the only dispersing offspring in the first brood carrying the altruism allele are half of the sons, resulting in a total of *n*/4 copies of the altruism allele in dispersing offspring. Although the sex of the dispersing offspring is biased toward males, the population is assumed to be so large that this small bias caused by a rare allele is of no consequence to the mating success of the males, and hence their reproductive value, and therefore, can be ignored. In the second brood, there are *n* offspring produced independently by the foundress and an additional *nb*/4 offspring produced with help from first‐brood daughters. In this brood, all offspring disperse, with half of the daughters and sons carrying the altruism allele, producing a total of *n*/2 + *nb*/8 copies of the allele in dispersing offspring. Therefore, from both broods, from the first cross, there is a total of 3*n*/4 + *nb*/8 copies of the allele in dispersing offspring (Table 1).

**Table 1:** Univoltine lifecycle: Haplodiploidy with a dominant altruism allele (*A*_1_)

Offspring	Proportion	No. helping	No. dispersing	No. *A*_1_ copies in dispersing
Cross 1: *A*_0_ *A*_1_ × *A*_0_				
1^st^ Brood (*n*)				
♀*A*_0_ *A*_0_	^1^/_4_	0	*n*/4	0
♀*A*_0_ *A*_1_	^1^/_4_	*n*/4	0	0
♂*A*_0_	^1^/_4_		*n*/4	0
♂*A*_1_	^1^/_4_		*n*/4	*n*/4
Total	1	*n*/4	3*n*/4	*n*/4
2^nd^ Brood (*n* + *nb*/4)				
♀*A*_0_ *A*_0_	^1^/_4_		*n*/4 + *nb*/16	0
♀*A*_0_ *A*_1_	^1^/_4_		*n*/4 + *nb*/16	*n*/4 + *nb*/16
♂*A*_0_	^1^/_4_		*n*/4 + *nb*/16	0
♂*A*_1_	^1^/_4_		*n*/4 + *nb*/16	*n*/4 + *nb*/16
Total	1		*n* + *nb*/4	*n*/2 + *nb*/8
Grand Total for Cross 1			7*n*/4 + *nb*/4	3*n*/4 + *nb*/8
Cross 2: *A*_0_ *A*_0_ × *A*_1_				
1^st^ Brood (*n*)				
♀*A*_0_ *A*_1_	^1^/_2_	*n*/2	0	0
♂*A*_0_	^1^/_2_		*n*/2	0
Total	1	*n*/2	*n*/2	0
2^nd^ Brood (*n* + *nb*/2)				
♀*A*_0_ *A*_1_	^1^/_2_		*n*/2 + *nb*/4	*n*/2 + *nb*/4
♂*A*_0_	^1^/_2_		*n*/2 + *nb*/4	0
Total	1		*n* + *nb*/2	*n*/2 + *nb*/4
Grand Total for Cross 2			3*n*/2 + *nb*/2	*n*/2 + *nb*/4
Weighted Mean				2*n*/3 + *nb*/6

*n* is brood size and *b* is the number of additional offspring produced by a foundress with help from a daughter.

With the cross in which the male carries the allele, all first‐brood daughters have the *A*_0_ *A*_1_ genotype and act as helpers (Table 1). The only dispersing offspring are sons, but none carry the altruism allele. In the second brood of this cross, all offspring disperse, and all daughters carry the altruism allele. Therefore, there is a total of *n*/2 + *nb*/4 copies of the allele in dispersing offspring from both broods. The two crosses occur with different probabilities due to the different number of genome copies carried by males and females. The first cross occurs with probability 2/3, and the second, with probability 1/3, equivalent to the different reproductive values of males and females due to haplodiploidy (Hamilton 1972). Averaged over the two crosses (weighted by their probabilities), the number of copies of a dominant altruism allele in dispersing offspring produced at the end of the breeding season is 2*n*/3 + *nb*/6 (Table 1).

However, we do not know the allele frequencies, since we have not specified a population size. Therefore, to determine the threshold amount of help, *b*, necessary for the altruism allele to increase in frequency, we compare the number of copies of the allele produced to the number that would have been produced if the allele had had no effect on helping behavior, that is, if the allele were neutral. This may be interpreted either as the relative change in allele frequency caused by the allele's expression or the change in allele frequency relative to the wild‐type allele (since a neutral *A*_1_ allele is indistinguishable from an *A*_0_ allele). With a neutral *A*_1_ allele, none of the daughters in the first brood act as helpers and all offspring disperse. It is clear from Table 1 that for the first cross there would be *n*/2 *A*_1_ allele copies in dispersing offspring in the first brood, and because there are no helpers, the second brood will have the same number. Therefore, for both broods of the first cross there is a total of *n A*_1_ allele copies in dispersing offspring. For the second cross, the number is the same, and therefore, averaged over the two crosses, the number of copies of a neutral *A*_1_ allele in dispersing offspring at the end of the breeding season is *n*.

Comparing the numbers of copies of the allele produced when it is expressed to the number produced when it is neutral gives the condition for which the altruism allele spreads in the population: *b* > 2. In inclusive fitness terms, using Hamilton's rule (Hamilton 1964a), from a helper's perspective she must cause the production of two additional offspring by the foundress, the helper's full siblings, on average related to her by ½, to meet the cost of having foregone being a dispersing, mated female who entered diapause, related to herself by 1. Pamilo (1991) used inclusive fitness accounting to derive this result.

##### Diploidy

With diploidy, there is a single cross to consider: *A*_0_ *A*_1_ × *A*_0_ *A*_0_. This gives the same result as the first cross with haplodiploidy: *A*_0_ *A*_1_ × *A*_0_. That is, there are 3*n*/4 + *nb*/8 copies of the dominant allele in dispersing offspring produced at the end of the breeding season with a univoltine lifecycle (Table 1). And, as with haplodiploidy, there are *n* neutral *A*_1_ allele copies produced. Therefore, the threshold condition for the spread of the allele with diploidy is also *b* > 2.

##### Haplodiploidy versus Diploidy

Comparing the two systems of ploidy shows that more copies of the allele are produce under haplodiploidy when *b* > 2. That is, for any amount of help above the threshold for the spread of the allele, more copies of the allele are produced with haplodiploidy than with diploidy. The number of altruism allele copies produced under haplodiploidy relative to the number produced under diploidy is:

$$R\;=\frac{4\left(4+b\right)}{3\left(6+b\right)}.$$

**Figure 2:**
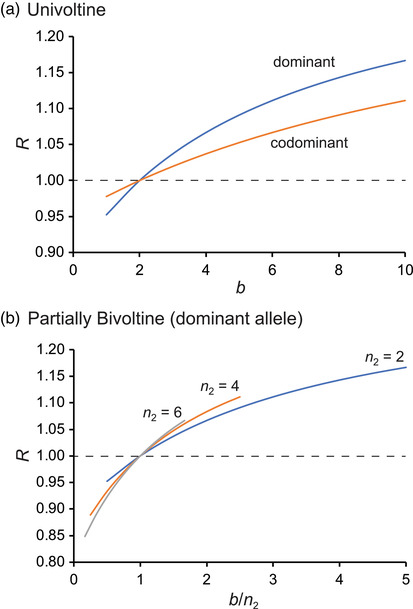
The number of allele copies produced under haplodiploidy relative to the number produced under diploidy, *R*, as a function of help, *b*, for a new altruism allele. **a**, Univoltine lifecycle; altruism allele dominance is indicated. Results for a codominant allele were derived in the same way as for a dominant allele (Supporting information Table S1). **b**, Partially bivoltine lifecycle with a dominant altruism allele and *b* ranging from 1 to 10; *n*_2_ is the second‐generation brood size.

For any *b* > 2, *R* > 1, and as *b* increases, *R* increases asymptotically to 4/3 (Fig. 2a). This asymptote is reached because there are 4/3 as many first‐brood altruist daughters produced under haplodiploidy as under diploidy due to all daughters inheriting the altruism allele from their father with haplodiploidy. For example, with a dominant allele, under diploidy half of first‐brood daughters are altruists, while under haplodiploidy, this proportion is the same when the foundress carries the allele, with probability 2/3, and 1 when the father carries the allele, with probability 1/3, giving an average of 2/3 of first‐brood daughters, which is 4/3 as many as with diploidy. Therefore, whenever *b* > 2, and thus reproductive altruism is favored, more copies of the altruism allele are produced with haplodiploidy than with diploidy.

#### Partially Bivoltine Lifecycle

The same approach was applied to the partially bivoltine lifecycle (Fig. 1). The main difference is that first‐brood daughters may either breed or help and all first‐brood sons breed. The second‐generation offspring, produced by first‐brood daughters and sons, disperse and mate and the daughters enter diapause, as do the daughters of the foundress’ second brood. Mating is assumed to be random in a very large population, and therefore, breeding first‐brood offspring mate with individuals carrying the wild‐type allele exclusively when the altruism allele is rare. First‐brood females that breed produce *n*_2_ offspring. Counting the numbers of alleles produced at the end of the breeding season, the threshold amount of help required for the altruism allele to spread is *b* > *n*_2_ for both dominant and codominant alleles under both haplodiploidy and diploidy (see Table 2 and Supporting information Tables S2 and S3). The inclusive fitness interpretation, using Hamilton's rule, is that because with monogamy siblings (on average) and offspring are equally related to a female, a first‐brood daughter must help her mother produce more offspring than the daughter would have produced on her own.

**Table 2:** Partially bivoltine lifecycle: Haplodiploidy with a dominant altruism allele (*A*_1_)

Offspring	Proportion	No. helping	No. dispersing	No. dispersing in generation 2	No. *A*_1_ copies in autumn dispersing
Cross 1: *A*_0_ *A*_1_ × *A*_0_					
1^st^ Brood (*n*)					
♀*A*_0_ *A*_0_	^1^/_4_	0	*n*/4	*nn*_2_/4	0
♀*A*_0_ *A*_1_	^1^/_4_	*n*/4	0	0	0
♂*A*_0_	^1^/_4_		*n*/4	*nn*_2_/4	0
♂*A*_1_	^1^/_4_		*n*/4	*nn*_2_/4	*nn*_2_/8
Total	1	*n*/4	3*n*/4	3*nn*_2_/4	*nn*_2_/8
2^nd^ Brood (*n* + *nb*/4)					
♀*A*_0_ *A*_0_	^1^/_4_		*n*/4 + *nb*/16		0
♀*A*_0_ *A*_1_	^1^/_4_		*n*/4 + *nb*/16		*n*/4 + *nb*/16
♂*A*_0_	^1^/_4_		*n*/4 + *nb*/16		0
♂*A*_1_	^1^/_4_		*n*/4 + *nb*/16		*n*/4 + *nb*/16
Total	1		*n* + *nb*/4		*n*/2 + *nb*/8
Grand Total for Cross 1					*nn*_2_/8 + *n*/2 + *nb*/8
Cross 2: *A*_0_ *A*_0_ × *A*_1_					
1^st^ Brood (*n*)					
♀*A*_0_ *A*_1_	^1^/_2_	*n*/2	0	0	0
♂*A*_0_	^1^/_2_		*n*/2	*nn*_2_/2	0
Total	1	*n*/2	*n*/2	*nn*_2_/2	0
2^nd^ Brood (*n* + *nb*/2)					
♀*A*_0_ *A*_1_	^1^/_2_		*n*/2 + *nb*/4		*n*/2 + *nb*/4
♂*A*_0_	^1^/_2_		*n*/2 + *nb*/4		0
Total	1		*n* + *nb*/2		*n*/2 + *nb*/4
Grand Total for Cross 2					*n*/2 + *nb*/4
Weighted Mean					*nn*_2_/12 + *n*/2 + *nb*/6

*n* is foundress brood size, *n*_2_ is the second‐generation brood size, and *b* is the number of additional offspring produced by a foundress with help from a daughter.

Comparing the two systems of ploidy, the number of altruism allele copies produced under haplodiploidy relative to the number produced under diploidy for a dominant allele is:

$$R\;=\frac{2\left(n_{2}+6+2b\right)}{3\left(n_{2}+4+b\right)}.$$

For any *b* > n_2_, *R* > 1, and as *b*/*n*_2_ increases, *R* increases asymptotically to 4/3 (Fig. 2b), as with the univoltine lifecycle. This equation gives the same result as for the univoltine lifecycle when *n*_2_ = 2. As *n*_2_ increases, the relative effect of haplodiploidy (*R*) also increases (Fig. 2b) because the threshold amount of help for the allele to spread increases and therefore haplodiploidy has more of an impact.

### NEIGHBOUR‐MODULATED FITNESS

It should be noted that these results cannot be derived by simply counting the dispersing offspring (and grand offspring with partial bivoltinism) of a mated pair. This neighbor‐modulated fitness would be calculated for a mated pair because although mating is random, the numbers of dispersing offspring depend on the genotypes of mated pairs. The reason this approach would not work is that there is a bias toward wild‐type alleles in dispersing first‐brood daughters, since those that carry the altruism allele are more likely to become helpers. For example, simply counting offspring gives the condition for the spread of the altruism allele with a univoltine lifecycle as *b* > 1, rather than the correct *b* > 2.

### PROBABILITY OF FIXATION OF A NEW ALTRUISM ALLELE AND ITS RATE OF SUBSTITUTION

**Figure 3:**
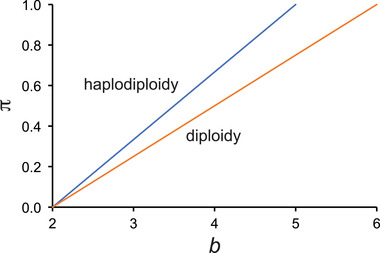
The probability of fixation a new dominant altruism allele under a univoltine lifecycle, π, as a function of the amount of help, *b*, for haplodiploidy and diploidy.

In a finite population, a new beneficial allele may have a very low probability of spreading to fixation because it is often lost when rare due to stochastic changes in allele frequencies (Haldane 1927). If the population size is large and selection is weak, the probability of fixation for a new beneficial allele is approximately twice the selective advantage of the heterozygote regardless of dominance or ploidy (Gillespie 2004, p. 93; Otto and Whitlock 2013). Therefore, for a heterozygote with relative fitness 1 + *s*, the probability of fixation of the beneficial allele is π≈2s. With a univoltine lifecycle, the relative fitness of the heterozygote is x/n, where *x* is the number of altruism alleles at the end of the breeding season and *n* is the fitness of the wild‐type homozygote (the number of “neutral” altruism alleles produced). Therefore, the selection coefficient is s=x/n−1. Plotting the probability of fixation, π, as a function of the amount of help, *b*, shows that with a univoltine lifecycle a new altruism allele has a higher probability of fixation under haplodiploidy than under diploidy for moderate amounts of help (2 < *b* < 6) (Fig. 3). A partially bivoltine lifecycle gives the same result when the second‐generation brood size is $n_{2}=\;2$.

However, it could be argued that the stronger selection of an altruism allele under haplodiploidy is irrelevant if the threshold amount of help favoring eusociality is the same under diploidy, because then, with recurrent mutation, eusociality will eventually evolve with diploidy under the same conditions. In this sense, haplodiploidy will have no impact on the evolution of eusociality on a time scale >1/ρ, where ρ=cNμπ is the rate of substitution of altruism alleles, where *c* is ploidy, *N* is the population size, and μ is the mutation rate for altruism alleles. For example, for a univoltine species with $N\;=10^{6}\;$ individuals and $\mu \;=10^{-9}\;$ mutations per amino acid site per generation, with haplodiploidy (c=1.5 genomes per individual on average with an even sex ratio) and b=3 (threshold b>2), π=0.33 and, thus, $1/\rho =\;2\times 10^{3}$ generations (years) per substitution. By comparison, with diploidy (c=2 genomes per individual) π=0.25 and, thus, $1/\rho =\;2\times 10^{3}$ generations per substitution also. Therefore, the greater number of genomes per individual for a diploid species, which attracts more mutations, cancels the effect of a higher probability of fixation of a new altruism allele for a haplodiploid species.

### FULL TIME COURSE ANALYSES

**Figure 4:**
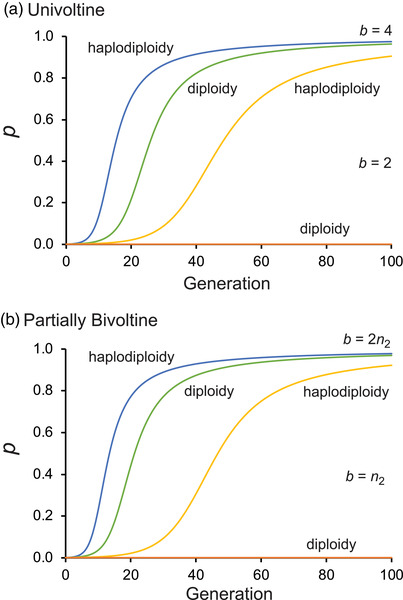
The full time course for the frequency of a dominant altruism allele, *p*. *b* is the amount of help provided by a first‐brood daughter. **a**, Univoltine lifecycle. **b**, Partially bivoltine lifecycle with second‐generation brood size *n*_2_ = 4.

The above results, from invasion analyses, were complimented by tracking the frequency of the altruism allele over annual breeding seasons using deterministic recursion equations, that is, assuming an infinite population size. The altruism allele spreads to fixation more quickly under haplodiploidy than under diploidy (Fig. 4). For diploidy, the threshold amount of help for the allele to spread is the same as derived by the invasion analyses above. However, for haplodiploidy, the threshold is lower. These results were confirmed using invasion analyses with the same conditions used with the recursion equations: the first‐brood sex ratio was adjusted to ensure an even sex ratio for dispersing offspring (Supporting information Tables S4 and S5). With a univoltine lifecycle, the threshold under haplodiploidy is *b* > 1.4, or 0.7 of the threshold under diploidy (*b* > 2). For a partially bivoltine lifecycle, the threshold under haplodiploidy is *b* > 0.7*n*_2_, compared to a threshold under diploidy of *b* > *n*_2_. Therefore, for both lifecycles, the threshold under haplodiploidy is 30% lower than the threshold under diploidy.

The reason for the lower threshold under haplodiploidy is that the first‐brood sex ratio is assumed to evolve so that the sex ratio of *dispersing* offspring remains even, resulting in a Fisherian optimal even sex ratio of reproducing individuals at the population level (West 2009). The result is that the overall first‐brood sex ratio becomes female biased when daughters act as helpers, which for a given proportion of females that are helpers generates more helpers, making haplodiploidy more effective in producing copies of the altruism allele in dispersing offspring at the end of the breeding season.

**Figure 5:**
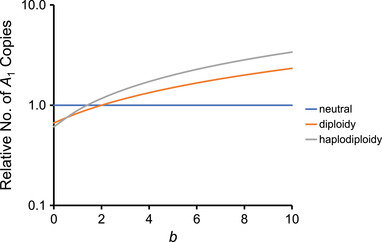
The number of copies of the altruism allele produced, relative to a neutral allele, as a function of the amount of help, *b*, when the first‐brood sex ratio is adjusted to ensure an even sex ratio of dispersing offspring. This shows that the threshold condition for the altruism allele to spread is *b* > 1.4 for haplodiploidy, and *b* > 2 for diploidy.

To explain this result further, the numbers of copies of the altruism allele, relative to a neutral allele, produced with diploidy and with haplodiploidy are shown for a univoltine lifecycle and a dominant altruism allele in Fig. 5 (based on Supporting information Table S4). The ratio of the number of copies of the altruism allele produced under haplodiploidy to the number produced under diploidy is

$$R\;=\frac{11+5b}{12+3b}.$$

*R* reaches an asymptote of 5/3. With diploidy, half of first‐brood daughters are altruists (*a* = ½) and, thus, the first‐brood sex ratio, in terms of the proportion of offspring that are female, is adjusted to $f\;=\frac{1}{2-a}\;=\frac{2}{3}\;$ to maintain an even sex ratio of dispersing offspring (1/3 of offspring are helpers, 1/3 are dispersing females, and 1/3 are dispersing males). Thus, with a new allele, 1/3 of all first‐brood offspring are altruist females (compared to ¼ without the sex ratio adjustment) under diploidy. The result is the same for haplodiploidy when the foundress carries the single copy of the altruism allele, which occurs with probability 2/3. When the male carries the allele, with probability 1/3, all first‐brood females are altruists (*a* = 1) and *f* = 1. Thus, on average, 5/9 of first‐brood offspring are altruist females under haplodiploidy, which is 5/3 more than with diploidy.

## Discussion

In one sense, haplodiploidy may favor the evolution of eusociality because invasion analyses show that there is stronger selection on an altruism allele under haplodiploidy than under diploidy. When a new altruism allele is favored, more copies of the allele are produced under haplodiploidy than under diploidy at the end of a breeding season. And, as shown for a univoltine lifecycle (and for a partially bivoltine lifecycle with $n_{2}=\;2$), for moderate amounts of help (2 < *b* < 6) this translates into a higher probability of fixation of a new dominant altruism allele. This level of help is consistent with the mean lifetime reproductive success reported for solitary, subsocial, and semisocial bees and wasps closely related to eusocial lineages. If lifetime reproductive success is assumed to be evenly spread over two broods, then the mean brood size for solitary, subsocial, and semisocial carpenter bees (*Xylocopinae*), which contain several primitively eusocial lineages (da Silva 2021), is four to five offspring (Danforth et al. 2019). For solitary potter wasps (*Eumeninae*), which are closely related to the eusocial vespid wasps (*Vespinae*) and paper wasps (*Polistinae*) (Piekarski et al. 2018), mean brood size is three to seven eggs laid (O'Neill 2001).

The mechanism underlying the stronger selection on an altruism allele under haplodiploidy is the transmission of the allele from a father to all his daughters. The result is a greater number of helpers under haplodiploidy than under diploidy, and thus, more copies of the allele being carried by dispersing offspring under haplodiploidy whenever helping produces more offspring than independent breeding. This is the same mechanism by which haplodiploidy favors eusociality in the protected invasion hypothesis (Reeve 1993). The hypothesis states that because in a monogamous species all daughters inherit any new mutation for alloparental care carried by their father, the mutation is less likely to be lost by genetic drift in a finite population if each alloparent increases colony output at least as much as an independently breeding female. However, as argued here, this does not translate into a higher rate of substitution for the allele under haplodiploidy since the higher probability of fixation of a new altruism allele under haplodiploidy is cancelled by the higher probability of the mutation arising in a diploid population.

The same mechanism explains why haplodiploidy favors eusociality in a more complex deterministic population genetic model by Fromhage and Kokko (2011), which examines the effects of mating system, colony dynamics, and demography in addition to ploidy. They argue that the transmission of an altruism allele from a father to all his daughters under haplodiploidy in a monogamous species increases the growth rate of the colony when reproductive altruism is favored. This in turn reduces the time to when the colony reaches its maximum size and begins producing reproductive individuals exclusively. Although this process may be valid, it does not seem to be relevant to the origin of eusociality, with small subsocial colonies and simple lifecycles.

A role for haplodiploidy in the origin of eusociality is revealed when considering the full time course of allele frequency dynamics. When analyzing the full time course, the effect of first‐brood daughters behaving as helpers on the sex ratio of dispersing first‐brood offspring must be considered because this sex ratio becomes progressively more male‐biased as the altruism allele increases in frequency. Assuming that selection acts to change the sex ratio of dispersing individuals toward the Fisherian optimum of equal numbers of males and females (West 2009), the first‐brood overall sex ratio was adjusted to ensure an even sex ratio of dispersing individuals. This adjustment makes the first‐brood sex ratio female‐biased whenever some daughters act as helpers, which in turn increases the proportion of offspring that are helpers, resulting in a 30% lower threshold amount of help for the spread of the altruism allele under haplodiploidy than under diploidy. This means that with a univoltine lifecycle and *b* = 2, or with a partially bivoltine lifecycle and *b* = *n*_2_ (the second‐generation brood size), an altruism allele is favored under haplodiploidy but not under diploidy. Interestingly, this is confirmed by much simpler invasion analyses incorporating the adjustment of the first‐brood sex ratio, indicating that such analyses are sufficient to predict the deterministic outcome of selection. Thus, when considering the evolution of the optimal sex ratio for dispersing offspring, haplodiploidy favors eusociality without a female‐biased sex ratio among dispersing offspring.

The prediction that the *overall* sex ratio of the first brood should be female‐biased in primitively eusocial species is supported by a well‐established first‐brood female bias in the primitively eusocial sweat bee (Halictinae) *Halictus rubicundus*, which exhibits brood bivalency, in which some first‐brood females become helpers while others mate and enter diapause (Yanega 1988; Yanega 1997). More generally, for 47 species and populations of sweat bees (Yanega 1997), there is a clear tendency for primitively eusocial species to have a first‐brood female bias compared to noneusocial species (Table 3; Chi‐square goodness‐of‐fit test: *df* = 1, χ^2^ = 21.496, *p* < 0.001).

**Table 3:** First‐brood overall sex ratio (counts) contingent on social organization for 47 species and populations of sweat bees (*Halictinae*) (Yanega 1997)

Social organization	Female bias	No female bias^b^
Primitively eusocial	29	1
Not eusocial^a^	6	11

^a^ Solitary, communal, or semisocial.

^b^ Unbiased or male biased.

It should be noted that other studies have proposed how haplodiploidy may favor eusociality through the manipulation of sex ratios. Haplodiploidy may reduce the fitness cost to the foundress of biasing her offspring sex ratio toward the helping sex, which is favored with local resource enhancement (Gardner and Ross 2013; Davies et al. 2016). This effect could enhance the female bias of the first brood. It has also been argued that the fitness cost of helping is lower for females under haplodiploidy when helpers can manipulate the nonhelper sex ratio (Rautiala et al. 2019). However, this hypothesis presupposes the existence of helpers. In addition, the sex of helpers in the Hymenoptera may be simply due to the preadaptation of female parental care (Ross et al. 2013; Davies et al. 2016).

Further tests of the mechanism proposed here by which haplodiploidy favors eusociality are not easily concieved since all Hymenoptera are haplodiploid. A possible test relies on the use of a species that is genetically polymorphic for eusociality, such as the sweat bee *Lasioglossum albipes*, which forms eusocial and noneusocial populations (Kocher et al. 2018). Crosses between eusocial and noneusocial populations could be used to establish colonies founded by either females or males carrying alleles for eusociality to determine the effects on the number of helpers and on the fitness of the mated pair. It may also be possible to compare haplodiploidy directly with diploidy by generating diploid males. Heterozygosity at the *csd* (*complimentary sex determiner*) locus causes individuals to develop as female, while homozygosity or hemizygosity (haploidy) causes individuals to develop as male (Beye et al. 2003; Heimpel and Boer 2008). Generating diploid males that are homozygous at *csd* may be possible through inbreeding even though such males are normally sterile (Cowan and Stahlhut 2004). Alternatively, the *fem* (*feminizer*) locus, which is involved in completing feminization through splice variants (Hasselmann et al. 2008), could be engineered to generate diploid males heterozygous at *csd*. Crosses with haploid and diploid males carrying alleles for altruism could then be compared to determine the threshold amounts of help necessary to favor eusociality.

The analyses presented here do not support the protected invasion hypothesis (Reeve 1993). Although a new allele for alloparental care has a higher probability of fixation under haplodiploidy with realistic levels of help, this effect is cancelled by the greater probability of the allele arising in a diploid population. Instead, the same underlying mechanism is responsible for a lower help threshold favoring eusociality under haplodiploidy than under diploidy when the sex ratio of dispersing first‐brood offspring remains even. The limited available data support the prediction of an overall first‐brood female bias in primitively eusocial species. This effect of haplodiploidy appears to have been overlooked because inclusive fitness accounting does not capture the effect of male haploidy on gene dynamics. The underlying genetics are important beyond determining relatedness since male haploidy affects group structure. Group selection approaches, however, will fail to capture the bias toward the wild‐type allele in dispersing first‐brood daughters. The gene‐centric approach taken here shows that haplodiploidy may favor eusociality in the absence of a female‐biased sex ratio in dispersing reproductive offspring.

## AUTHOR CONTRIBUTIONS

The sole author (J.d.S) concieved and designed the study, and carried out the analyses and wrote the paper.

## DATA ARCHIVING

computer codes are archived in Zenodo https://doi.org/10.5281/zenodo.6510980

Associate Editor: H. Helantera

Handling Editor: A.G. McAdam

## Supporting information

Supporting Information
