# Supplementary material for: Gene dynamics of haplodiploidy favor eusociality in the Hymenoptera
Source: Evolution. 2022 Jun 6;76(7):1546–55. doi: 10.1111/evo.14518 (PMC9543898; doi:10.1111/evo.14518)
Supplement: Supplementary file 1 — Supporting Information [file EVO-76-1546-s001.pdf]

Recursion Equations

First-brood sex ratio in fitness calculations

Because some first-brood females may act as helpers, depending on their genotype, the sex ratio of first-brood *dispersing* offspring would be biased toward males if the brood adult sex ratio is even. In the extreme case, if all first-brood females acted as helpers, then all dispersing offspring would be male. Assuming equal costs of producing sons and daughters, it is further assumed that the population sex ratio of dispersing first-brood offspring evolves to remain even (West 2009). That is, *on average* females produce first-brood sex ratios that result in an even sex ratio in dispersing offspring. This is implemented most easily by assuming that each female produces a first-brood sex ratio that results in an even sex ratio of dispersing offspring. This sex ratio, in terms of the proportion of offspring that are female, is $f = \frac{1}{2-a}$, where a is the proportion of first-brood females that act as helpers. For example, if $a = \frac{1}{2}$, then $f = \frac{2}{3}$ and thus $af = \frac{1}{3}$ of offspring are female helpers, $f - af = \frac{1}{3}$ are dispersing females and $1 - f = \frac{1}{3}$ are dispersing males.

Univoltine lifecycle

Fitness

In the present context, fitness is a property of mated pairs of individuals, rather than genotypes, because with fertility selection, although mating is random, gamete fusions are not. With a univoltine lifecycle, fitness consists of two components: the number of offspring produced in the first brood that disperse and the number produced in the second brood, all of which disperse. The first-brood fitness component is $n - afn$, where n is the number of first-brood offspring produced by the foundress. The second-brood fitness component is the number offspring produced independently by the foundress plus additional offspring produced with help from first-brood daughters that do not disperse: $n + afnb$, where b is the additional number of offspring produced with help from each first-brood daughter. Because the sex ratio of dispersing offspring is even in every case, the different reproductive values of males and females due to haplodiploidy cancel out when calculating relative fitness and may be ignored. That is, each mated pair, consisting of a male and female, always produces equal numbers of male and female offspring that disperse and mate randomly with individuals from the population to form new mated pairs. Therefore, the fitness of a mated pair with genotypes i and j is:

$$W_{ij} = \left(n - a_{ij}f_{ij}n + n + a_{ij}f_{ij}nb\right)/2$$

Note that a_{ij} is a function of the genotypes of a mated pair, as is f_{ij} because it is a function of a_{ij}. The right-hand side of the equation is divided by 2 because each dispersing offspring mates randomly in the population to produce a new mated pair. Mean fitness is the sum of the products of mated-pair fitness and the frequency of the mated pair, assuming random mating, over all possible mated pairs:

$$\bar{W} = \sum_{i=0}^{2}\sum_{j=0}^{1} W_{ij} x_i y_j$$

where x_i and y_j are the genotype frequencies of females and males, respectively (with the value of the subscript indicating the number of altruism allele copies carried by a diploid female or a haploid male). However, because the genotypes of dispersing first-brood females are biased against those carrying the altruism allele, fitnesses of mated pairs are not sufficient to determine allele dynamics. Genotype frequencies must be tracked to determine the fate of the altruism allele.

Recursion equations

Deterministic recursion equations were used to track genotype frequencies over generations. The recursion equations for haplodiploidy are described in detail here; the recursion equations for diploidy differ only in that diploid male genotypes must be indexed. Recursion equations are derived separately for females and males because of the genotype bias in dispersing first-brood females and because of the ploidy difference with haplodiploidy. To calculate the frequency of genotype k in females in the next spring generation, we must know the proportion of genotype k in first-brood daughters that disperse, $d^f_{k(1),ij}$, which is a function of genotypes i and j of a mated pair. The proportion of genotype k in second-brood daughters is given by $d^f_{k(2),ij}$ (this proportion does not reflect any genotype bias since all second-brood offspring disperse). The overall proportion of females with genotype k in dispersing females in both broods is the sum of the proportions from each brood weighted by the proportion of fitness contributed by the brood of origin. The proportion of fitness contributed by the first brood is given by $v_{ij(1)}$. Therefore, the overall proportion of females with genotype k in dispersing offspring of parents with genotypes i and j is

$$d^f_{k,ij} = d^f_{k(1),ij} v_{ij(1)} + d^f_{k(2),ij}\left(1 - v_{ij(1)}\right)$$

Thus, the frequency of genotype k in females in the next spring generation, after random mating in the current spring generation (formation of mated pairs), is

$$x'_k = \frac{1}{\bar{W}} \sum_{i=0}^{2}\sum_{j=0}^{1} W_{ij} x_i y_j d^f_{k,ij}$$

Since sexes are considered individually and sex ratios are even, both fitness and mean fitness are halved, which cancels out.

For males, the frequency of genotype l in the next generation is

$$y'_l = \frac{1}{\bar{W}} \sum_{i=0}^{2}\sum_{j=0}^{1} W_{ij} x_i y_j d^m_{l,i}$$

where $d^m_{l,i}$ is the proportion of males with genotype l produced by a female with genotype i, which is the same for both broods. Allele frequencies for the entire population, including

both males and females, are calculated from the sex-specific genotype frequencies, taking into consideration that females are diploid and males are haploid.

Partially bivoltine lifecycle

Fitness

With a bivoltine lifecycle fitness also consists of two components. The first component is the number of offspring produced by first-brood dispersing offspring, that is, the second generation, all of which disperse. The second component is the number of offspring produced in the second brood of the first generation, all of which also disperse. Dispersing first-brood offspring mate at random in the population and produce $(n_1 - afn_1)\, n_2/2$ offspring, where n_1 is the number of offspring produced independently (without help) by the foundress in each first-generation brood, and n_2 is the number of offspring produced independently by each dispersing first-brood offspring as part of a mated pair, forming the second generation. These brood sizes may differ ($n_2 < n_1$) since first-brood dispersing females may experience higher mortality due to dispersal and mating and must establish new nests. The second-generation brood size is halved because these offspring are the product of outbreeding and are therefore evenly allocated to the descendants of the focal mated pair and mates chosen at random from the population. The second brood produced by the founding mated pair is the same as for a univoltine lifecycle. Therefore, the fitness of a mated pair is:

$$W_{ij} = \left[\left(n_1 - a_{ij}f_{ij}n_1\right)\frac{n_2}{2} + n_1 + a_{ij}f_{ij}n_1 b\right]/2$$

Recursion equations

To use recursion equations to track genotype frequencies across generations we must determine the genotype proportions of offspring in the second generation. These proportions depend on the proportions of genotypes of dispersing first-brood offspring, which depend on the genotypes of the mated pair. The genotype proportions in the second generation also depend on the population-wide genotype frequencies of the dispersing first-brood's random mates in the population. Second-generation females are produced by both dispersing first-brood females and males. The proportion of genotype k in second-generation females produced by dispersing first-brood daughters of parents with genotypes i and j is:

$$g^f_{k(2),ij\,(daughters)} = \sum_{i=0}^{2}\sum_{j=0}^{1}\sum_{l=0}^{2}\sum_{m=0}^{1} h^f_{k,lm} d^f_{l(1),ij} d^m_{m(1)}$$

where $h^f_{k,lm}$ is the proportion of genotype k in females produced by a dispersing first-brood daughter with genotype l mated with a male with genotype m, $d^f_{l(1),ij}$ is the proportion of genotype l in dispersing first-brood daughters of parents with genotypes i and j, and d^m_m is the population-wide frequency of genotype m in first-brood males. This latter frequency is calculated as

$$d^m_{m(1)} = \frac{1}{\overline{W}_{(1)}} \sum_{i=0}^{2} \sum_{j=0}^{1} W_{ij(1)} x_i y_j \, d^m_{m,i}$$

where $W_{ij(1)}$ is the first-brood fitness of a mated pair with genotypes i and j, which is the same as the component of fitness due to the first brood with a univoltine lifecycle, $\overline{W}_{(1)}$ is the mean first-brood fitness of mated pairs, x_i and y_j are the genotype frequencies of the mated pair, and $d^m_{m,i}$ is the proportion of males with genotype m produced by a female with genotype i.

The proportion of genotype k in second-generation females produced by dispersing first-brood sons is:

$$g^f_{k(2),ij\ (sons)} = \sum_{i=0}^{2} \sum_{j=0}^{1} \sum_{l=0}^{2} \sum_{m=0}^{1} h^f_{k,lm} h^m_{m,i} d^f_{l(1)}$$

where $h^m_{m,i}$ is the proportion of genotype m in dispersing first-brood sons of mothers with genotype i, and $d^f_{l(1)}$ is the population-wide frequency of genotype l in first-brood females. The latter frequency is calculated as

$$d^f_{l(1)} = \frac{1}{\overline{W}_{(1)}} \sum_{i=0}^{2} \sum_{j=0}^{1} W_{ij(1)} x_i y_j d^f_{l(1),ij}$$

The proportions of genotype k in second-generation females produced by dispersing first-brood daughters and sons are averaged since dispersing first-brood sons and daughters occur in equal proportions: $g^f_{k(2),ij}$.

The proportion of genotype k in dispersing females in autumn is the sum of the proportions of the genotype in first-generation (second-brood) and second-generation females, each weighted by the proportion of fitness contributed by their respective generation:

$$g^f_{k,ij} = g^f_{k(2),ij} u_{ij(2)} + g^f_{k(1),ij} \left(1 - u_{ij(2)}\right)$$

where $g^f_{k(1),ij}$ is the proportion of genotype k in the first generation, which is equivalent to the proportion in the second brood in the case of univoltinism, and $u_{ij(2)}$ is the proportion of fitness contributed by the second generation. Then, the frequency of genotype k in females in the next spring generation, after random mating in the current spring generation, is

$$x'_k = \frac{1}{\overline{W}} \sum_{i=0}^{2} \sum_{j=0}^{1} W_{ij} x_i y_j g^f_{k,ij}$$

With haplodiploidy, second generation males are produced only by dispersing first-brood daughters. Therefore, the proportion of genotype k in second-generation males is:

$$g^{m}_{k(2),ij} = \sum_{i=0}^{2}\sum_{j=0}^{1}\sum_{l=0}^{2} h^{m}_{k,l} d^{f}_{l(1),ij}$$

And the proportion of genotype k in dispersing males in the autumn is the sum of the proportions of the genotype in first-generation (second-brood) and second-generation males, each weighted by the proportion of fitness contributed by their respective generation:

$$g^{m}_{k,ij} = g^{m}_{k(2),ij} u_{ij(2)} + g^{m}_{k(1),ij}\left(1 - u_{ij(2)}\right)$$

where $g^{m}_{k(1),ij}$ is the proportion of genotype k in the first generation, which is equivalent to the proportion in the second brood in the case of univoltinism. Then, the frequency of genotype k in males contributing to the next spring generation is

$$y'_k = \frac{1}{\overline{W}} \sum_{i=0}^{2}\sum_{j=0}^{1} W_{ij} x_i y_j g^{m}_{k,ij}$$

References

West, S. A. 2009. Sex Allocation. Princeton University Press, Princeton.

Table S1. Univoltine lifecycle: Haplodiploidy with a codominant altruism allele (A_1).

Offspring	Proportion	No. Helping	No. Dispersing	No. A_1 Copies in Dispersing
Cross 1: A_0A_1 x A_0				
1st Brood (n)				
♀ A_0A_0	¼	0	$n/4$	0
♀ A_0A_1	¼	$n/8$	$n/8$	$n/8$
♂ A_0	¼		$n/4$	0
♂ A_1	¼		$n/4$	$n/4$
Total	1	$n/8$	$7n/8$	$3n/8$
2nd Brood ($n + nb/8$)				
♀ A_0A_0	¼		$n/4 + nb/32$	0
♀ A_0A_1	¼		$n/4 + nb/32$	$n/4 + nb/32$
♂ A_0	¼		$n/4 + nb/32$	0
♂ A_1	¼		$n/4 + nb/32$	$n/4 + nb/32$
Total	1		$n + nb/8$	$n/2 + nb/16$
Grand Total for Cross 1			$15n/8 + nb/8$	$7n/8 + nb/16$
Cross 2: A_0A_0 x A_1				
1st Brood (n)				
♀ A_0A_1	½	$n/4$	$n/4$	$n/4$
♂ A_0	½		$n/2$	0
Total	1	$n/4$	$3n/4$	$n/4$
2nd Brood ($n + nb/4$)				
♀ A_0A_1	½		$n/2 + nb/8$	$n/2 + nb/8$
♂ A_0	½		$n/2 + nb/8$	0
Total	1		$n + nb/4$	$n/2 + nb/8$
Grand Total for Cross 2			$7n/4 + nb/4$	$3n/4 + nb/8$
Weighted Mean				$5n/6 + nb/12$

n is brood size and b is the number of additional offspring produced with help from a daughter. With a codominant altruism allele, a first-brood daughter carrying one copy has a 0.5 probability of acting as a helper.

Table S2. Partially bivoltine lifecycle: Haplodiploidy with a neutral altruism allele (A_1).

Offspring	Proportion	No. Helping	No. Dispersing	No. Dispersing in Generation 2	No. A_1 Copies in Autumn Dispersing
Cross 1: A_0A_1 x A_0					
1st Brood (n)					
♀ A_0A_0	¼	0	$n/4$	$nn_2/4$	0
♀ A_0A_1	¼	0	$n/4$	$nn_2/4$	$nn_2/8$
♂ A_0	¼		$n/4$	$nn_2/4$	0
♂ A_1	¼		$n/4$	$nn_2/4$	$nn_2/8$
Total	1	0	n	nn_2	$nn_2/4$
2nd Brood (n)					
♀ A_0A_0	¼		$n/4$		0
♀ A_0A_1	¼		$n/4$		$n/4$
♂ A_0	¼		$n/4$		0
♂ A_1	¼		$n/4$		$n/4$
Total	1		n		$n/2$
Grand Total for Cross 1					$nn_2/4 + n/2$
Cross 2: A_0A_0 x A_1					
1st Brood (n)					
♀ A_0A_1	½	0	$n/2$	$nn_2/2$	$nn_2/4$
♂ A_0	½		$n/2$	$nn_2/2$	0
Total	1	0	n	nn_2	$nn_2/4$
2nd Brood (n)					
♀ A_0A_1	½		$n/2$		$n/2$
♂ A_0	½		$n/2$		0
Total	1		n		$n/2$
Grand Total for Cross 2					$nn_2/4 + n/2$
Weighted Mean					$nn_2/4 + n/2$

n is foundress brood size, n_2 is the second-generation brood size, and b is the number of additional offspring produced with help from a daughter.

Table S3. Partially bivoltine lifecycle: Haplodiploidy with a codominant altruism allele (A_1).

Offspring	Proportion	No. Helping	No. Dispersing	No. Dispersing in Generation 2	No. A_1 Copies in Autumn Dispersing
Cross 1: A_0A_1 x A_0					
1st Brood (n)					
♀ A_0A_0	¼	0	$n/4$	$nn_2/4$	0
♀ A_0A_1	¼	$n/8$	$n/8$	$nn_2/8$	$nn_2/16$
♂ A_0	¼		$n/4$	$nn_2/4$	0
♂ A_1	¼		$n/4$	$nn_2/4$	$nn_2/8$
Total	1	$n/8$	$7n/8$	$7nn_2/8$	$3\ nn_2/16$
2nd Brood ($n + nb/8$)					
♀ A_0A_0	¼		$n/4 + nb/32$		0
♀ A_0A_1	¼		$n/4 + nb/32$		$n/4 + nb/32$
♂ A_0	¼		$n/4 + nb/32$		0
♂ A_1	¼		$n/4 + nb/32$		$n/4 + nb/32$
Total	1		$n + nb/8$		$n/2 + nb/16$
Grand Total for Cross 1					$3\ nn_2/16 + n/2 + nb/16$
Cross 2: A_0A_0 x A_1					
1st Brood (n)					
♀ A_0A_1	½	$n/4$	$n/4$	$nn_2/4$	$nn_2/8$
♂ A_0	½		$n/2$	$nn_2/2$	0
Total	1	$n/4$	$3n/4$	$3\ nn_2/4$	$nn_2/8$
2nd Brood ($n + nb/4$)					
♀ A_0A_1	½		$n/2 + nb/8$		$n/2 + nb/8$
♂ A_0	½		$n/2 + nb/8$		0
Total	1		$n + nb/4$		$n/2 + nb/8$
Grand Total for Cross 2					$nn_2/8 + n/2 + nb/8$
Weighted Mean					$nn_2/6 + n/2 + nb/12$

n is foundress brood size, n_2 is the second-generation brood size, and b is the number of additional offspring produced with help from a daughter. With a codominant altruism allele, a first-brood daughter carrying one copy has a 0.5 probability of acting as a helper.

Table S4. Univoltine lifecycle with first-brood sex ratio adjustment: Haplodiploidy with a dominant altruism allele (A_1).

Offspring	Proportion	No. Helping	No. Dispersing	No. A_1 Copies in Dispersing
Cross 1: A_0A_1 x A_0				
1st Brood (n; a = ½; f = 2/3)				
♀ A_0A_0	1/3	0	$n/3$	0
♀ A_0A_1	1/3	$n/3$	0	0
♂ A_0	1/6		$n/6$	0
♂ A_1	1/6		$n/6$	$n/6$
Total	1	$n/3$	$2n/3$	$n/6$
2nd Brood ($n + nb/3$)				
♀ A_0A_0	¼		$n/4 + nb/12$	0
♀ A_0A_1	¼		$n/4 + nb/12$	$n/4 + nb/12$
♂ A_0	¼		$n/4 + nb/12$	0
♂ A_1	¼		$n/4 + nb/12$	$n/4 + nb/12$
Total	1		$n + nb/3$	$n/2 + nb/6$
Grand Total for Cross 1			$5n/3 + nb/3$	$2n/3 + nb/6$
Cross 2: A_0A_0 x A_1				
1st Brood (n; a = 1; f = 1)				
♀ A_0A_1	1	n	0	0
♂ A_0	0			
Total	1	n	0	0
2nd Brood ($n + nb$)				
♀ A_0A_1	½		$n/2 + nb/2$	$n/2 + nb/2$
♂ A_0	½		$n/2 + nb/2$	0
Total	1		$n + nb$	$n/2 + nb/2$
Grand Total for Cross 2			$n + nb$	$n/2 + nb/2$
Weighted Mean				$11n/18 + 5nb/18$

n is brood size, b is the number of additional offspring produced with help from a daughter, f is the first-brood sex ratio and a is the proportion of first-brood daughters that are altruists.

Table S5. Partially bivoltine lifecycle with first-brood sex ratio adjustment: Haplodiploidy with a dominant altruism allele (A_1).

Offspring	Proportion	No. Helping	No. Dispersing	No. Dispersing in Generation 2	No. A_1 Copies in Autumn Dispersing
Cross 1: A_0A_1 x A_0					
1st Brood (n; a = ½; f = 2/3)					
♀ A_0A_0	1/3	0	$n/3$	$nn_2/3$	0
♀ A_0A_1	1/3	$n/3$	0	0	0
♂ A_0	1/6		$n/6$	$nn_2/6$	0
♂ A_1	1/6		$n/6$	$nn_2/6$	$nn_2/12$
Total	1	$n/3$	$2n/3$	$2nn_2/3$	$nn_2/12$
2nd Brood ($n + nb/3$)					
♀ A_0A_0	¼		$n/4 + nb/12$		0
♀ A_0A_1	¼		$n/4 + nb/12$		$n/4 + nb/12$
♂ A_0	¼		$n/4 + nb/12$		0
♂ A_1	¼		$n/4 + nb/12$		$n/4 + nb/12$
Total	1		$n + nb/3$		$n/2 + nb/6$
Grand Total for Cross 1					$nn_2/12 + n/2 + nb/6$
Cross 2: A_0A_0 x A_1					
1st Brood (n; a = 1; f = 1)					
♀ A_0A_1	1	n	0	0	0
♂ A_0	0				
Total	1	n	0	0	0
2nd Brood ($n + nb$)					
♀ A_0A_1	½		$n/2 + nb/2$		$n/2 + nb/2$
♂ A_0	½		$n/2 + nb/2$		0
Total	1		$n + nb$		$n/2 + nb/2$
Grand Total for Cross 2					$n/2 + nb/2$
Weighted Mean					$nn_2/18 + n/2 + 5nb/18$

n is foundress brood size, n_2 is the second-generation brood size, b is the number of additional offspring produced with help from a daughter, f is the first-brood sex ratio and a is the proportion of first-brood daughters that are altruists.
